# Anti-cervical carcinoma effect of *Portulaca oleracea L*. polysaccharides by oral administration on intestinal dendritic cells

**DOI:** 10.1186/s12906-019-2582-9

**Published:** 2019-07-05

**Authors:** Rui Zhao, Xingyue Shao, Guiyan Jia, Yulan Huang, Zhe Liu, Bocui Song, Jinzuo Hou

**Affiliations:** 1 Department of Pharmaceutical Engineering, College of Life Science & Biotechnology, Heilongjiang August First Land Reclamation University, Daqing High-Tech Industrial Development Zone, Daqing, 163319 People’s Republic of China; 2 Department of Gynaecology and Obstetrics, Daqing Oilfield Hospital, Daqing, 163311 People’s Republic of China

**Keywords:** *Portulaca oleracea* L*.* polysaccharide, Intestinal immune, Dendritic cells, Apoptosis, Signaling pathway

## Abstract

**Background:**

Cervical cancer is the second most prevalent cancer worldwide. *Portulaca oleracea* L. polysaccharide (POL-P3b) has been found to have enhancing immune and anti-cervical cancer activity by oral administration. Dendritic cells (DC) play a key roles in regulating intestinal immune homeostasis. In this study, we analyzed the inhibition apoptosis effects of POL-P3b on intestinal DC and relevant mechanisms.

**Methods:**

Intestinal DC was isolated from U14-bearing mice treated with POL-P3b (50 mg/kg, 100 mg/kg and 200 mg/kg, respectively). The effects of POL-P3b on proliferation and inhibiting apoptosis in intestinal DC were evaluated by MTT assay, Hoechst 33342 and Annexin V-FITC/PI staining. Mitochondrial Ca^2+^ was analyzed using flow cytometry instrument. The potential mechanisms underlying POL-P3b-induced protection of intestinal DC from cervical cancer-induced apoptosis were detected with Western blotting evaluation of expression levels of TLR4 and relevant proteins for apoptotic signaling pathway.

**Results:**

We found that a large number of intestinal DC were apoptosis in U14-bearing mice. Treatment with POL-P3b in U14-bearing mice at different doses for 12 d resulted in a significant increase in intestinal DC survival, and the mechanisms were related to inhibiting DC apoptosis.

**Conclusion:**

Our results suggested that POL-P3b-induced protection against tumor-induced intestinal DC apoptosis through stimulating the TLR4-PI3K/AKT-NF-κB signaling pathway. This study enhanced understanding of the oral administration with POL-P3b exerted on anti-tumor activity and its action mechanism.

## Background

Cervical cancer is the most common gynecological malignancy. Each year, more than 500.000 new cases are diagnosed with cervical carcinoma, mostly in developing countries [1]. Cytotoxic chemotherapy is an effective treatment for cervical cancer, especially in patients with recurrent metastasis. Unfortunately, chemical anti-tumor agents have a large toxic side effect and are easy to develop drug resistance in long-term application [2]. Therefore, science researchers have showed a great interest in Natural Chinese herbal medicine as anti-tumor drugs. Herbal active ingredients have a remarkable effect on the treatment of cervical cancer, and low toxicity to normal tissue.

*Portulaca oleracea* L. is a kind of wild vegetable with high nutritional value and medicinal value. Modern medical investigations have showed that *Portulaca oleracea* L. has antibacterial effect and possesses certain inhibitory effect on dysentery bacillus, typhoid bacillus, *Escherichia coli* and *Staphylococcus aureus*. It is known as “natural antibiotics” with non-toxic side effects [3, 4]. Our previous researchs had clearly displayed that the polysaccharide (POL-P3b), an active ingredient isolated and purified from *Portulaca oleracea* L., possessed the activity of inhibiting cervical carcinoma by oral delivery, and the mechanisms were associated with enhancing the body’s immune [4]. The immune response to tumor antigen is the result of the interaction among antigen presenting cells (APC), T cells and B cells. Dendritic cells (DC) are the most powerful professional APC in the body, which could efficiently absorb, process and deliver antigen. Immature DC has strong migration ability, and mature DC can effectively activate the initial T cells, which are at the center of initiation, regulation and maintenance of immune response, including anti-tumor immunity [5, 6]. DC are also important immune cells in the intestinal immune system. In recent years, a growing interest in the topic that dietary nutrients affect systemic immune through the intestine [7, 8]. Tumor cells could modify their own surface antigen and change the microenvironment of tumor tissues to affect the function of immune cells, so as to escape the body’s immune recognition and attack [9]. It is generally proposed that DC apoptosis could be an important factor by which tumor cells might escape immune system surveillance [10]. Studies have showed that tumor cells could cause functional defects and decreased number of DC, and also induce DC apoptosis. The number of DC is closely related to the prognosis and survival for tumor patients [11]. It has been recently demonstrated that polysaccharide improve survival of immune cells such as DC [12]. So far, the effect of POL-P3b on intestinal DC survival and the detailed mechanisms responsible for the anti-cervical carcinoma activity by oral administration remain unclear. The aims of this study were to investigate the possible mechanisms of POL-P3b for the protection of intestinal DC from tumor-induced apoptosis in gut. The experimental results might provide scientific and comprehensive evidence for POL-P3b as an oral natural antitumor agent.

## Methods

### Materials

The whole plant of *Portulaca oleracea* L. used in the study was collected in July in Heilongjiang province, China. The experimental material was authenticated by Dr. Sun Yue-Chun at Heilongjiang BaYi Land Reclamation University, where the herbarium voucher has been kept (ID: 334473). Isolation, purification and identification of POL-P3b were based on our previous published work [4]. *Escherichia coli* LPS, mitomycin C, MTT and Hoechst 33342 were purchased from Sigma Chemical Co.. Recombinant mouse GM-CSF, IL-4 and IL-2 were purchased from R&D Systems; mouse CD11c, MicroBeads and MS separation column was purchased from Miltenyi Biotec; FITC-conjugated Annexin V was purchased from PharMingen, San Diego, CA and propidium iodide (PI) was purchased from Sigma Chemical Co.. Antibodies against Bcl-2, cytochrome c and caspase 3 were from Santa Cruz Biotechnology, Inc.(Santa Cruz, CA). Ab against phospho-ERK (p-ERK), ERK, phospho-p38 (p-p38), p38, phospho-JNK (p-JNK) and JNK were purchased from Cell Signaling (Beverly, MA USA). LY294002 (an PI3K kinase inhibitor), was purchased from Sigma-Aldrich (St. Louis, MO, USA).

### Cell lines and culture

The mouse cervical carcinoma U14 cells were obtained from the Cell Bank of Institute of Basic Medical Sciences (Peking Union Medical College, Beijing, China). Cells were cultured in DMEM supplemented with 10% FBS and 1% penicillin-streptomycin at 37 °C in a humidified atmosphere containing 5% CO_2_. The medium was changed 2–3 times each week.

### Establishment and grouping of tumor mouse models

Female Kunming mice (18–22 g) were purchased from the Experimental Animal Center of Hayida Medical University. The experimental mice were housed under a suitable temperature (23 ± 3 °C) and humidity (55 ± 15%) with a 12 h light/dark cycle. The mice were allowed free access to clean water and diet.

To analysis the anti-tumor efficacy of POL-P3b in vivo, U14 cells (1 × 10^7^/mL) were s.c. injected into mice. After 24 h, all the experimental mice were randomly divided into four groups including model group (sterile water), low dose of POL-P3b (50 mg/kg), medium dose of POL-P3b (100 mg/kg) and high dose of POL-P3b (200 mg/kg). All mice were continuously treated for 12 days. On day 13 of the experiment, mice were anesthetized using 2.5% isoflurane, and sacrificed by exsanguination. The transplanted tumors of mice were harvested and weighed.

### Cell proliferation assay

Mice were humanely killed by cervical dislocation, and the small intestine and the ileum was taken out, then the membrane and fat was removed. The specific experimental methods for isolation and culture of intestinal CD11c + DCs were according to our previous literature [13]. The intestinal DC was placed in 96-well culture plates (1.0 × 10^4^ cells per well). The cell concentration was determinated by MTT assay. Briefly, cells were seeded in a 96-well plate (2 × 10^5^ cells/mL) and cultivated for 24 h. Cells were then incubated with fresh medium containing different dose of POL-P3b for 48 h. Then, MTT (0.5 mg/mL) was added to the medium, and cells were further incubated for 4 h at 37 °C, following lysis with DMSO. The absorbance was measured at 570 nm using a microplate ELISA reader (Model 550, Bio-Rad, USA).

### Annexin V assay

Apoptosis was detected by measuring phosphatidyl serine exposure and membrane permeability. Intestinal DC was harvested and double-stained with FITC-conjugated Annexin V and propidium iodide (PI). Samples were analysed by the FACScan flow cytometer with Cell Quest software (Becton-Dickinson, Franklin Lakes, NJ, USA).

### Morphological study with fluorescence microscopy

Apoptosis morphology was evaluated using Hoechst 33342 fluorescence staining. Intestinal DC was seeded in chamber slides. The cells were fixed in 3% paraformaldehyde for 20 min and stained with 10 μg/mL of Hoechst 33342 at 37 °C in the dark for 20 min. The results were observed by fluorescence microscope (OLYMPUS, CKX41-F32FL, Japan).

### Measurement of mitochondrial Ca^2+^ content

Mitochondrial Ca^2+^ content of intestinal DC was measured using flow cytometer, and the procedure in this study was a modification of the method. To get 0.1 mL 1 × 10^6^ cells/mL single cell suspension, and then 10% chicken red blood cells were added as internal standard. 1 mL Fluo3 was added into cell suspension at 37 °C in the dark for 30 min. After PBS washing, the Ca^2+^ content of mitochondrial was immediately analyzed using flow cytometry instrument.

### Western blot analysis

The protein expression was assessed using western blot technique. The protein concentration was determined by the Bradford method. The lysate was separated by 10% SDS-PAGE and transferred to a nitrocellulose membrane blocked with 5% nonfat milk. Different kinds of protein were detected using specific primary antibodies with the final concentration of 2.5 μg/mL overnight at 4 °C. After washing three times with TBST buffer, the membrane was incubated with the secondary antibodies. The intensity of the specific immunoreactive bands were visualized by enhanced chemiluminescence (ECL).

### Statistical analysis

Results were expressed as the mean ± S.D. Statistical evaluation was performed by a one-way analysis of variance (ANOVA) with Dunnett’s test. *P* < 0.05 were considered significant.

## Results

### Effects of oral POL-P3b on tumor inhibition rate of U14-bearing mice

To evaluate the antitumor activity of POL-P3b in vivo, we first established the cervical carcinoma model by s.c. injection of U14 cells into mice. After treatment with POL-P3b for 12 days, no adverse event was found in any of the experimental groups. The tumor weight of U14-bearing mice in the POL-P3b (M) and POL-P3b (H) groups was significantly lower than that of the model group (*P* < 0.05, *P* < 0.01, respectively). The inhibition rate of tumor in the POL-P3b (L), POL-P3b (M) and POL-P3b (H) groups was 5.23, 25.07 and 46.56%, respectively (Table 1).

**Table 1:** Effect of POL-P3b by oral administration on tumor growth and weight of U14-bearing mice ($\overline{x}$±S.D)

Groups	Animal number		Body weight (g)		Tumor weight (g)	Inhibition rate (%)
	Beginning	End	Beginning	End		
Control	10	10	20.37 ± 1.69	25.55 ± 1.86	–	–
Model	10	10	20.35 ± 1.34	23.17 ± 1.41	3.63 ± 0.71	–
POL-P3b(L)	10	10	20.84 ± 1.51	22.23 ± 1.60	3.44 ± 0.26	5.23
POL-P3b(M)	10	10	21.06 ± 1.87	21.31 ± 1.03	2.72 ± 0.54^*^	25.07
POL-P3b(H)	10	10	20.79 ± 1.71	21.35 ± 1.51	1.94 ± 1.12^**^	46.56

Compared with model, ^*^P < 0.05, ^**^P < 0.01. The tumor inhibition rate was expressed according to the following formula: (the mean tumor weight of control group - the mean tumor weight of treated group)/ the mean tumor weight of control group × 100

### Effects of oral POL-P3b on intestinal DC survival of U14-bearing mice

**Fig. 1:**
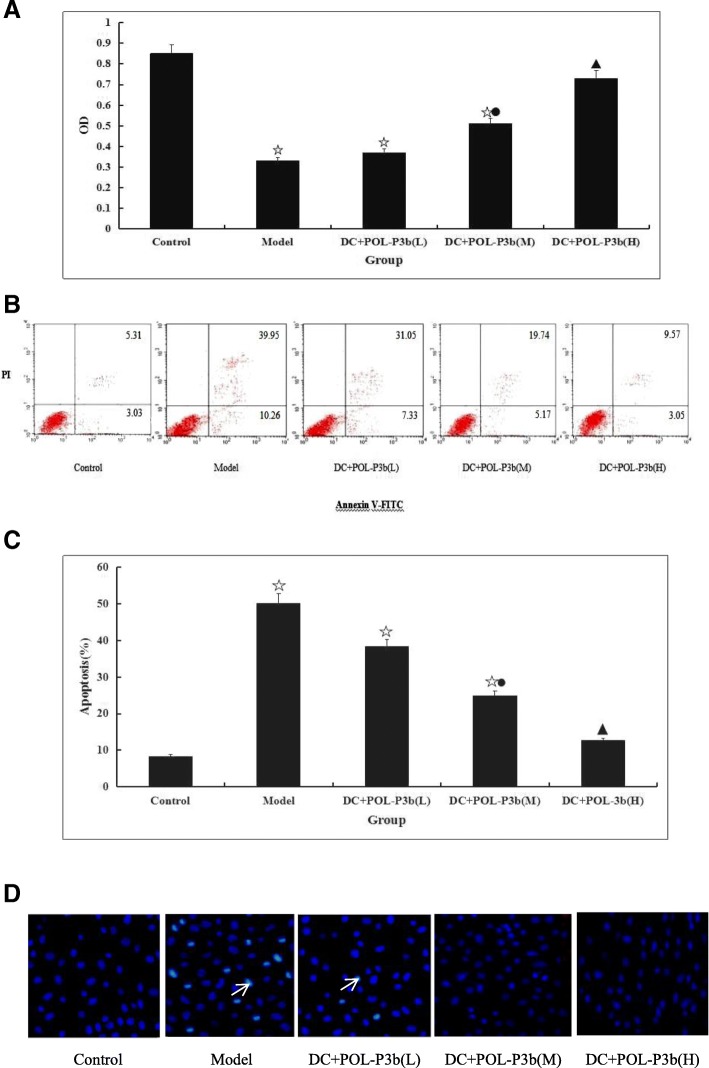
POL-P3b induced the proliferation and inhibited apoptosis of intestinal DC by oral administration **a** The cell viability was evaluated by MTT assay. Data are mean values of triplicate assays ±SD, compared with control, ^☆^*P* < 0.01; compared with model, ^●^*P* < 0.05, *P* < 0.01. **b** Annexin V-FITC/PI staining was used to analyze apoptosis. **c** Representative results and summative data from three independent experiments were shown. Each bar represents means ± SD (*n* = 3). compared with control, ^☆^*P* < 0.01; compared with model, ^●^*P* < 0.05, *P* < 0.01. **d** Intestinal DCs isolated from mice treated with POL-P3b (50 mg/kg,100 mg/kg and 200 mg/kg, respectively) for 12 d were stained with Hoechst 33342 (blue)

Since it has been showed early that polysaccharide might prolong DC survival in cultures (12), we determine the cell proliferation effects of POL-P3b on intestinal DC of U14-bearing mice by MTT assay. As showed in Fig. 1a, the proliferation of intestinal DC in model mice were significantly decreased compared with control group (*P* < 0.01). However, the proliferation was increased in the treatment group of POL-P3b, and the effect was obvious in high dose group (*P* < 0.01).

To determine whether POL-P3b protect intestinal DC from apoptosis, we assessed the intestinal DC viability by Annexin V assay. As showed in Fig. 1b, c, intestinal DC apoptosis in model group was signifcantly increased, with 50.21% of apoptotic rate, compared with the control group (8.34%). However, apoptotic rate presented markedly lower in the POL-P3b group (38.38, 24.91 and 12.62%, respectively), compared with the model group. Apoptosis is a process of programmed cell death characterized by biochemical and morphological indicators. To further confirm the inhibiting apoptosis effect of POL-P3b on intestinal DC, morphological observation was performed by Hoechst 33342 staining. As depicted in Fig. 1d, the apoptotic features were obvious in model group, such as, cell shrinkage, rounding up and enhanced blue fluorescence intensity (showed by arrows). The apoptotic intestinal DC amount was reduced after oral POL-P3b administration.

### Effect of oral POL-P3b on mitochondrial [Ca^2+^] of intestinal DC

**Fig. 2:**
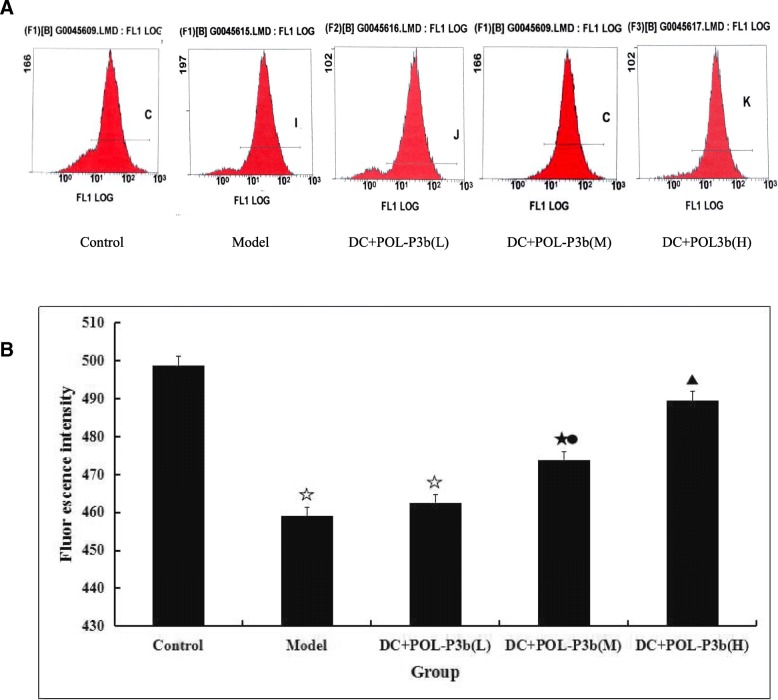
Effect of POL-P3b on mitochondrial Ca^2+^ of intestinal DC by oral administration **a** Mitochondrial Ca^2+^ was analyzed using flow cytometry instrument. Data were collected in FSC (forward scatter) and SSC (side scatter) and a total of 10,000 events were collected for each sample. **b** The level of Ca^2+^ was indicated with fluorescence intensity (I). The computation formula is as follows: I = Log (x-mode) × 340. Data were expressed as mean ± S.D. (*n* = 10). compared with control, *P* < 0.05, ^☆^*P* < 0.01; compared with model, ^●^*P* < 0.05, *P* < 0.01

To investigate the mechanism of oral POL-P3b for the protection of intestinal DC from tumour induced apoptosis, the content of mitochondrial Ca^2+^, an important regulator for mitochondrial function were evaluated. Our result showed that the content of Ca^2+^ in model group was significantly decreased compared with the control group (*P* < 0.01). While in the oral POL-P3b treatment groups, the content of Ca^2+^ was significantly higher than that in the model group (*P* < 0.05, *P* < 0.01) (Fig. 2).

### Oral POL-P3b-induced protection for intestinal DC from cervical carcinoma-induced apoptosis involved in TLR4-PI3K/AKT-NF-κB signaling pathway

**Fig. 3:**
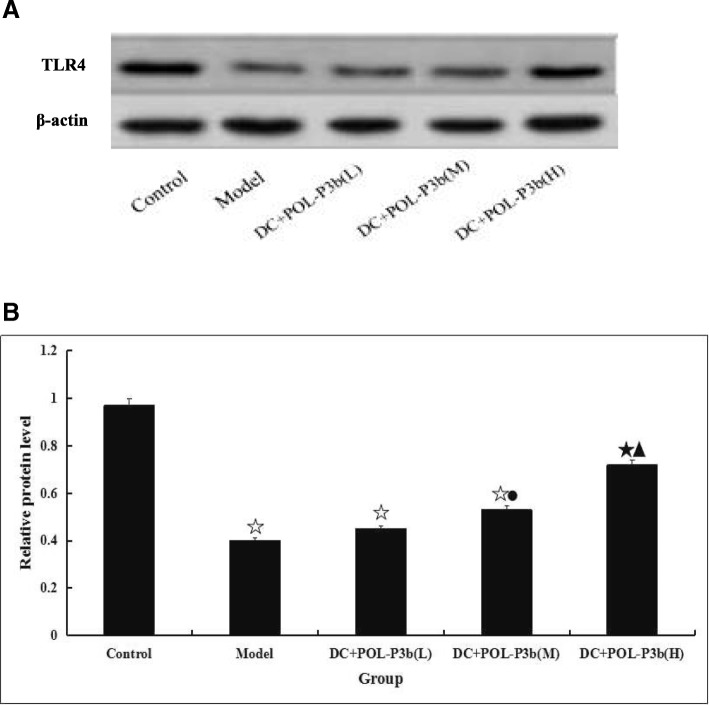
The expression of TLR4 and β-actin in intestinal DC **a** Western blot analysis showing the expression of TLR4 in intestinal DC isolated from U14-bearing mice treated with oral POL-P3b. **b** Statistical bar graph of expression of TLR4 protein. Immunoblots were scanned within the linear range and quantitated using the computer software. The quantitated values represent the mean ± S.D. compared with control, *P* < 0.05, ^☆^*P* < 0.01; compared with model, ^●^*P* < 0.05, *P* < 0.01

**Fig. 4:**
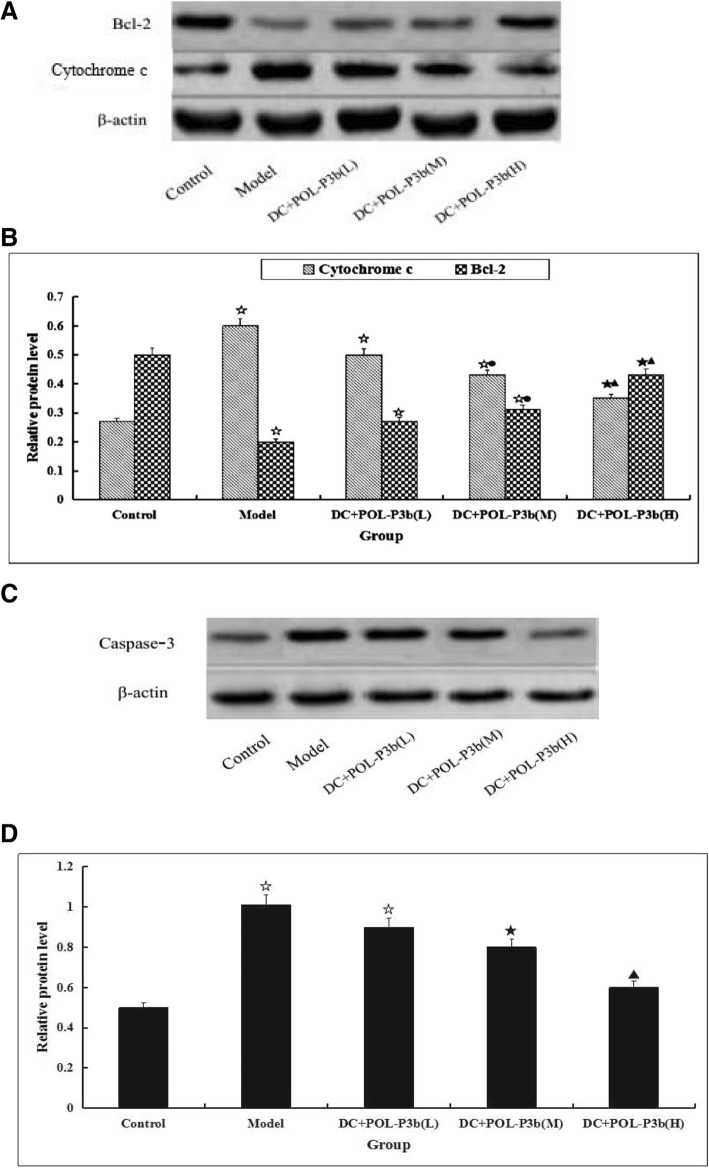
Effect of POL-P3b by oral administration on intrinsic apoptotic-related proteins in intestinal DC **a** Expression of Bcl-2 and cytosolic Cytochrome c in intestinal DC was analyzed by western blot in three independent experiments. **c** Expression of caspase-3 in DC were analyzed by western blot in three independent experiments. **b** and **d** Statistical bar graph of the protein expression. Immunoblots were scanned within the linear range and quantitated using the computer software. The quantitated values represent the mean ± S.D. compared with control, *P* < 0.05, ^☆^*P* < 0.01; compared with model, ^●^*P* < 0.05, *P* < 0.01

The above experimental results had demonstrated that oral POL-P3b could inhibit tumor by decreasing apoptosis of intestinal DC isolated from U14-bearing mice. In the next paragraphs, the more detail mechanisms were discussed. We first detected the stimulative activities of oral POL-P3b on TLR4 expression in intestinal DC. As showed in Fig. 3, the TLR4 expression was decreased in model group, however, POL-P3b treatment increased the level of TLR4. Furthermore, we investigated the involvement of the mitochondrial mediated intrinsic suppressive apoptotic pathway by assessing the release of cytochrome c from the mitochondria into the cytoplasm, and the expression of the downstream proteins. As showed in Fig. 4, a significant decrease of cytochrome c release and caspase-3 activity were observed in POL-P3b groups. While the content of Bcl-2 was significantly higher in POL-P3b group than that in the model group. The results suggested that POL-P3b inhibit apoptosis in intestinal DC isolated from U14-bearing mice at a concentration-dependent manner and the phenomenon occurred through the activation of the intrinsic mitochondrial apoptotic pathway.

**Fig. 5:**
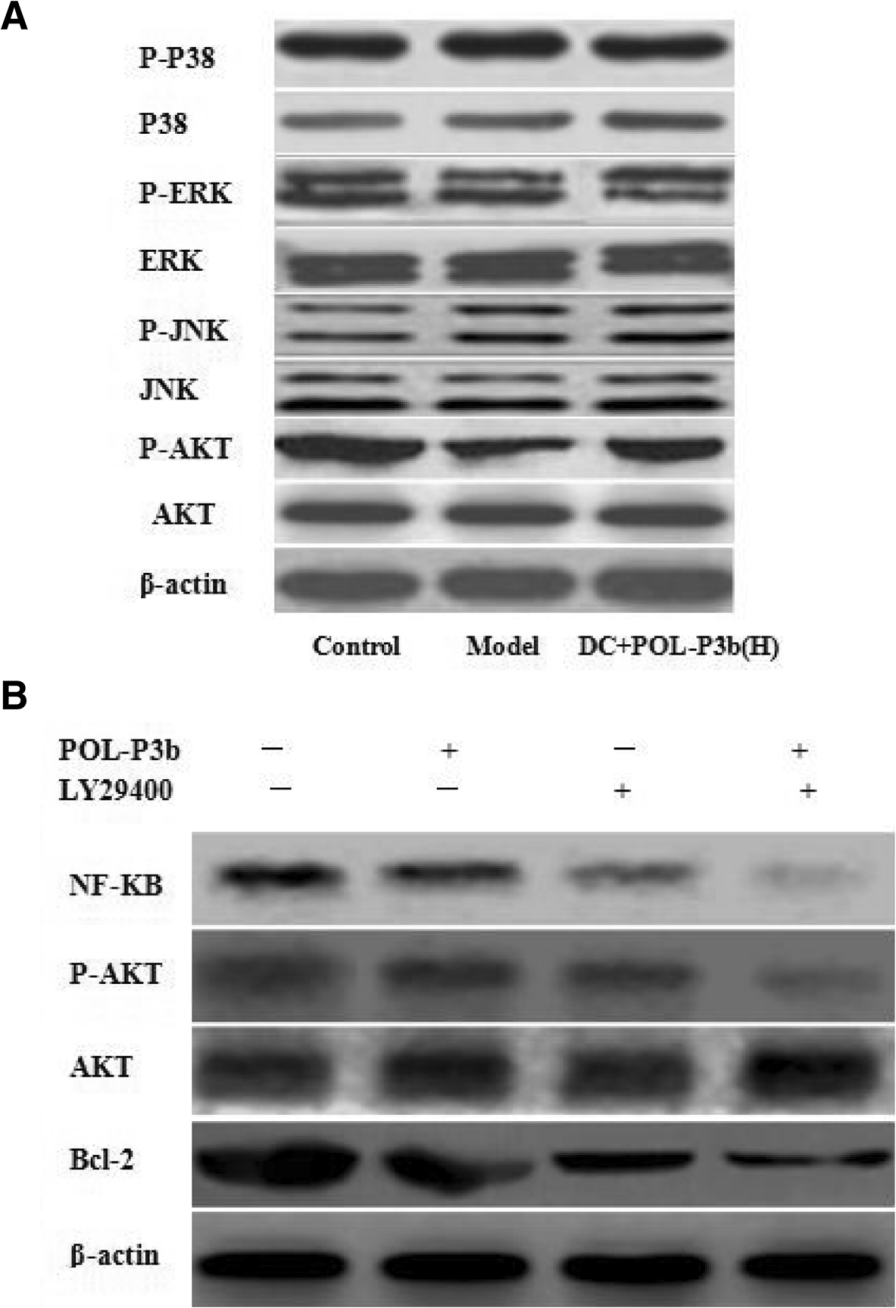
Activity of POL-P3b-induced protection of intestinal DC from cervical cancer-induced apoptosis involved in PI3K/AKT and NF-κB signaling pathway **a** Western blot analysis showing the levels of phosphorylated p38, ERK, JNK and AKT in intestinal DC. **b** The PI3K downstream target was analyzied. Intestinal DC isolated from mice treated with or without POL-P3b were pretreated by LY294002 (60 μmol/L) for 1.5 h. The Akt activity, the NF-κB activation and the expression of Bcl-2 in intestinal DC was suppressed by the pretreatment of LY294002

Stimulation of TLR4 usually leads to activation of multiple intracellular signaling pathways including PI3K/AKT and MAPK [14]. According to the above-mentioned results, we selected the best dose for the subsequent experiments. In the current study, oral POL-P3b treatment increased the phosphorylation of AKT, however, POL-P3b had no appreciable effects on p-p38, p-ERK and p-JNK (Fig. 5a). To adequately confirm PI3K/AKT signaling pathway was responsible for oral POL-P3b-induced protection against tumor-induced intestinal DC apoptosis, we used PI3K kinase inhibitors, such as LY294002. The downstream Akt target is NF-κB, an essential transcription factor that up-regulates multiple survival genes in many types of cells. Our results showed that Akt and NF-κB activity or the expression of Bcl-2 in intestinal DC was suppressed by the treatment of LY294002 (Fig. 5b). Importantly, on the basis of inhibitors, POL-P3b could further inhibit the Akt, NF-κB activity and Bcl-2 expression in intestinal DC. Therefore, these results demonstrated that POL-P3b-induced protection against tumor-induced intestinal DC apoptosis through the TLR4-PI3K/AKT-NF-κB signaling pathway.

## Discussion

Polysaccharides are the major component of medicinal herb and nutritional food, and have various biological activities, including antioxidant, anti-tumor and immunomodulation [15]. Accumulating evidence has indicated that the anti-tumor effects of polysaccharides are not because of their cancer cytotoxicity but their stimulatory effects on effector activation, such as DC, which result in powerful anti-tumor immune responses [16]. DC are the most potent APC in priming naive T cells and maintaining specific immune responses against tumors [17]. Induction of DC apoptosis is one of the escape mechanisms for tumor cells from the immune surveillance system. The gastrointestinal tract represents an important entry site for oral drugs. Studies have showed that DC derived from intestinal mucosa are more potent in stimulating allogeneic T-cells proliferation rather than DC from spleen [18]. POL-P3b, an indigestible polysaccharide with a high molecular weight, was difficultly diffused through plasma membrane. It was necessary to clarify the mechanisms that oral anti-tumor agent might rely on the intestinal immune system to activate the systemic immune response. In this study, we proved the facts that apoptosis was increased in intestinal DC of U14-bearing mice. However, the intervention with POL-P3b by oral administration was effective for protecting intestinal DC from apoptosis and promoting intestinal DC survival in U14-bearing mice in a dose-response manner. Our results suggested that after oral administration with POL-P3b, the anti-tumor activity was stimulated by promoting the proliferation of intestinal DC and inhibiting its apoptosis, which further confirmed the relationship between the activity of polysaccharides by oral delivery and the intestinal immune function.

DC could pick up antigen and transport across the intestinal epithelium through various different routes, such as MyD88-dependent signal through TLRs [19]. Polysaccharide-related receptors mainly include TLR2 and TLR4 [20]. Stimulation of TLR4 has been associated with the initiation of both apoptotic and antiapoptotic pathways, the balance of which determines the outcome of immune responses including innate and adaptive response [21]. Here, we investigated the possible mechanism of POL-P3b by oral administration for the protection of intestinal DC from carcinoma induced apoptosis. In this present study, it was found that TLR4 participated in the inhibiting apoptosis effect of POL-P3b on intestinal DC isolated from tumor-burdened mice.

The activation of NF-κB is a necessary condition for DC to stimulate T cell activation. And T cells are stimulated by DC can be strengthened through extending duration of NF-κB activation state. The additional loss of mitochondrial Ca^2+^ further indicates the mitochondrial role for inducing the apoptosis related to NF-κB inhibition. NF-κB inhibits cell apoptosis by inducing and up-regulating Bcl-2 family proteins, as well as blocking the upstream activation of caspase-3 [22]. The specific binding site of NF-κB is found in the caspase 3 promoter, and NF-κB directly up-regulates the expression of Bcl-2 by the transcriptional pathway [23]. We assumed that TLR4 signaling usurped through intestinal DC of U14-bearing mice, triggered tumor self-protection mechanisms and then led to immune escape. In agreement with these findings, we showed here that oral POL-P3b induced the increase of Bcl-2 and mitochondrial Ca^2+^. In addition to this, POL-P3b prevented cytochrome c release from mitochondria and reduced the expression of caspase 3.

**Fig. 6:**
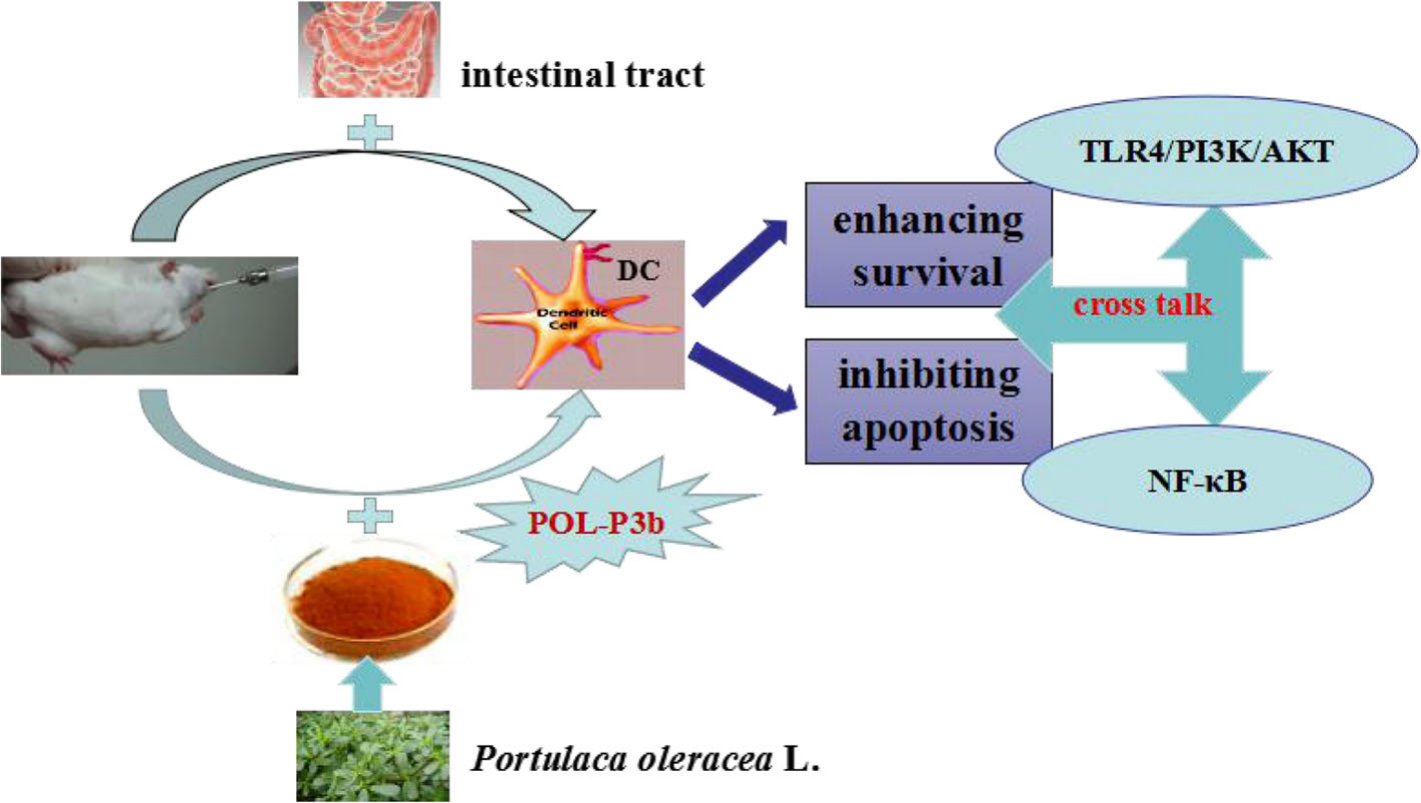
POL-P3b-induced protection against tumor-induced intestinal DC apoptosis through the cross talk between TLR4/PI3K/AKT and NF-κB signaling pathway

We continued to evaluate the downstream signaling pathway of TLR4. Several different immunological relevant signaling elements, including PI3K/AKT and ERK/JNK/p38 MAPKs phosphorylation were measured to assess the activation of these kinases for intestinal DC of U14-bearing mice treated with oral POL-P3b. We found that POL-P3b treatment stimulated NF-κB relevant protein phosphorylation. AKT phosphorylation was signifcantly increased following oral POL-P3b treatment. To amply confirm the mediating role of PI3K/AKT in signaling, chemical inhibitors LY294002 was used to block NF-κB activation and decreased the expression of Bcl-2 and p-AKT. Our data revealed that oral POL-P3b-induced protection against tumor-induced intestinal DC apoptosis through PI3K/AKT signaling pathway, which regulate many immunological relevant proteins (Fig. 6).

## Conclusions

POL-P3b could inhibit the growth of cervical carcinoma by oral administration and the mechanism was related to inducing protection against tumor-induced intestinal DC apoptosis through stimulating the TLR4/PI3K/AKT-NF-κB signaling pathway. This study enhanced understanding that POL-P3b may be used as a dietary agent for strengthening immunity against tumor.

## Data Availability

The datasets used and/or analysed during the current study available from the corresponding author on reasonable request.

## Abbreviations

APC: Antigen-presenting cells
DC: Dendritic cells
ECL: Enhanced chemiluminescence
HPSEC: High-performance size exclusion chromatography
MLNs: Mesenteric lymph nodes
MTT: 3-(4,5-dimethylthiazol-2-yl)-2,5-diphenyl-2H-tetrazolium bromide
MW: Molecular weight
POL-P3b: *Portulaca oleracea* L. polysaccharide
